# Structural investigations of T854A mutation in EGFR and identification of novel inhibitors using structure activity relationships

**DOI:** 10.1186/1471-2164-16-S5-S8

**Published:** 2015-05-26

**Authors:** Sukriti Goyal, Salma Jamal, Asheesh Shanker, Abhinav Grover

**Affiliations:** 1 Department of Bioscience and Biotechnology, Banasthali University, Tonk, Rajasthan, India - 304022; 2 School of Biotechnology, Jawaharlal Nehru University, New Delhi, India - 110067

## Abstract

**Background:**

The epidermal growth factor receptor (EGFR) is a member of the ErbB family that is involved in a number of processes responsible for cancer development and progression such as angiogenesis, apoptosis, cell proliferation and metastatic spread. Malfunction in activation of protein tyrosine kinases has been shown to result in uncontrolled cell growth. The EGFR TK domain has been identified as suitable target in cancer therapy and tyrosine kinase inhibitors such as erlotinib have been used for treatment of cancer. Mutations in the region of the EGFR gene encoding the tyrosine kinase (TK) domain causes altered responses to EGFR TK inhibitors (TKI). In this paper we perform molecular dynamics simulations and PCA analysis on wild-type and mutant (T854A) structures to gain insight into the structural changes observed in the target protein upon mutation. We also report two novel inhibitors identified by combined approach of QSAR model development.

**Results:**

The wild-type and mutant structure was observed to be stable for 26 ns and 24 ns respectively. In PCA analysis, the mutant structure proved to be more flexible than wild-type. We developed a 3D-QSAR model using 38 thiazolyl-pyrazoline compounds which was later used for prediction of inhibitory activity of natural compounds of ZINC library. The 3D-QSAR model was proved to be robust by the statistical parameters such as r^2 ^(0.9751), q^2^(0.9491) and pred_r^2^(0.9525).

**Conclusion:**

Analysis of molecular dynamics simulations results indicate stability loss and increased flexibility in the mutant structure. This flexibility results in structural changes which render the mutant protein drug resistant against erlotinib. We report two novel compounds having high predicted inhibitory activity to EGFR TK domain with both wild-type and mutant structure.

## Introduction

The epidermal growth factor receptor (EGFR) is a member of the ErbB family that is involved in a number of processes responsible for cancer development and progression such as angiogenesis, apoptosis, cell proliferation and metastatic spread [1]. The EGFR family comprises of four receptors namely, EGFR (ErbB1/HER1), ErbB2 (HER2/neu), ErbB3 (HER3) and ErbB4 (HER4). Various mechanisms including gene amplification and mutations result in a disturbed regulatory mechanism of EGFR signalling [2]. Malfunction in activation of such kinases has been shown to result in uncontrolled cell growth. The EGFR TK domain has been identified as suitable target in cancer therapy and drugs such as erlotinib have been used for treatment of cancer. However, mutations in the region of the EGFR gene encoding the tyrosine kinase (TK) domain causes altered responses to EGFR TK inhibitors (TKI) [2]. In 2004, these mutations were first identified in patients with non-small cell lung cancer (NSCLC)[1]. NSCLC of Caucasian origin account for 15% while NSCLC of Asian ethnicity for 30% of EGFR mutations. These mutations are known to be associated with non-smoking status, adenocarcinoma histology and female gender [3,4].

The most common mutations involve point mutations in exon 18 and exon 21, insertions or deletions in exon 19, insertions/duplications and point mutations in exon 20 [5]. Destabilization of equilibrium between the active and inactive state of EGFR kinase activity toward promoting enzyme activation is a result of these mutations which in turn causes EGFR to translate into tumor growth and gives a survival advantage [1,6]. Mutations T790M (gatekeeper), M766T (C helix), L718A (solvent chanel) and T854A (activation loop) are most common in erlotinib resistance [7]. For this study we will be studying a drug-sensitive second-site EGFR mutation, T854A, which occurs due to change of Guanine in place of Adenine at nucleotide 2560 (exon 21)[8]. This non-synonymous single nucleotide polymorphism (SNP) results in substitution of Alanine for Threonine at position 854. The T854A residue is located at the bottom of the ATP binding site on C-lobe and its side chain is in contact distance of erlotinib or gefitinib. Thus, T854A substitution results in loss of contacts and binding affinity to these inhibitors.

*In silico *methodology for drug development is a viable and good option when compared to conventional drug development methods. One such *in silico *method involves development of quantitative structure activity relationship (QSAR) which establishes a correlation between the structure and inhibitory activity of molecular fragments of interests. 3D-QSAR is a robust technique in drug design process used to predict the inhibitory activities of the prospective lead compounds by applying the knowledge of three-dimensional properties of the lead compounds through a chemometric approach [9,10]. It develops models which indicate the synthesis of novel inhibitors assuming that the receptor binding ability is related to its inhibitory activity [11,12]. For development of QSAR model, the binding site of receptor is considered to be rigid and that the ligand molecules belong to a set of congeneric series [12]. Molecular fields including hydrophobic, steric and electrostatic interaction energies are calculated for the set of compounds. A molecular field analysis model is generated and evaluated for its robustness by calculation of statistical parameters.

In this study we performed molecular dynamics simulations on both wild-type (WT) and mutant (T854A) structures and analysed the structural changes [13-16]. A 3D-QSAR model was developed using 38 thiazolyl-pyrazoline derivatives reported by Lv et al (2011) against WT EGFR [17]. This model was then used to screen ZINC libraries for compounds with high predicted activity values which can be considered as lead drug candidates against both WT and mutant (T854A). This paper gives insights to the structural changes brought about by single nucleotide polymorphism in tyrosine kinase domain of EGFR. The compounds reported in this study can be considered for further experimental validation as potent lead compounds.

## Materials and methods

### Generation of wild-type and mutant EGFR structures

The crystal structure of WT EGFR was extracted from Protein Data Bank (PDB) [PDB ID: 4G5J][18]. The obtained crystal structure was first prepared using Protein preparation utility of Schrodinger [19-21]. Mutant structure was generated using Schrodinger Glide software through Maestro interface. The WT structure of EGFR was also subject to the identical *in silico *mutational method in which the wild-type residue (Threonine) was mutated to itself (Threonine) to ensure that mutation process is uniform for all structures. These structures were then subject to MD simulations.

### Molecular dynamics simulations

The GROMACS package [22,23] was used for carrying out MD simulations using the same conditions as mentioned in previous works of our lab [15,24,25]. Comparative analysis of structural deviations in WT and mutated (T854A) structures of EGFR were carried out using Gromacs utility tools such as g_rmsf, g_rms, g_gyrate, g_sas etc.

### Principal component analysis

The essential dynamics (ED) method was used for the computation of eigenvectors and eigenvalues with their projection along the first two principal components with the help of a protocol present in the GROMACS software package [26]. The principal component analysis (PCA) or ED is a protocol that simplifies the complexity of data obtained and extracts the important motion in MD that are significant for the biological function of the protein [26]. In this evaluation, a covariance matrix was created from the obtained trajectories once the rotational and translational movements were removed. The matrix was then diagonalized to identify a set of eigenvectors and eigenvalues. The amplitude of eigenvectors and the displacement of atoms along each of these eigenvectors show the concentrated motions of protein along each direction and were represented by eigenvalues. This analysis was performed using g_covar and g_anaeig of GROMACS utility tools.

### Selection and presentation of data set for QSAR model development

**Figure 1:**
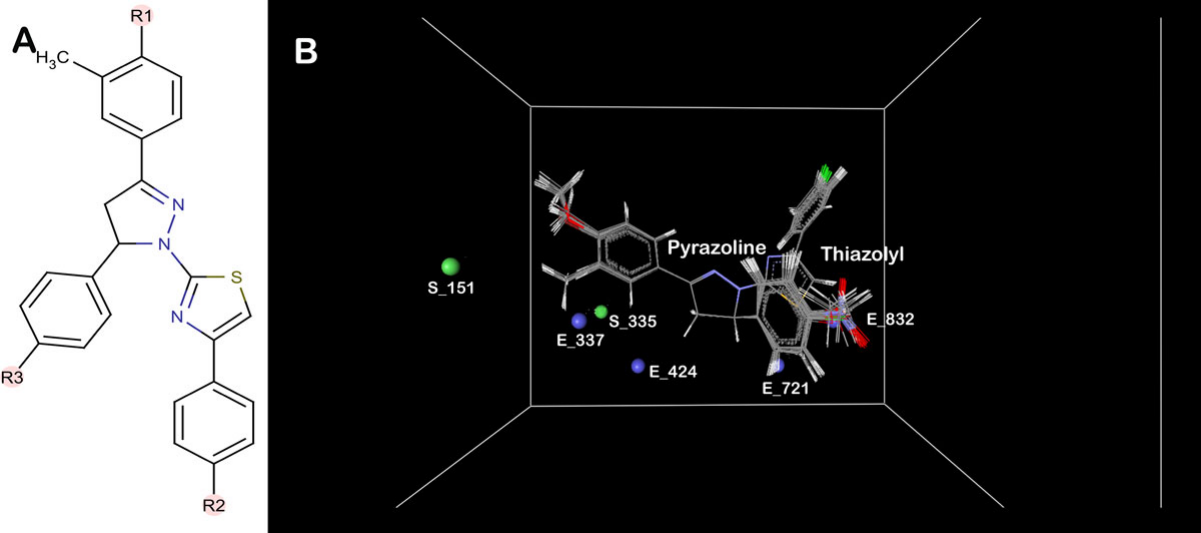
**(a) Representation of structure of common template of thiazolyl-pyrazoline compounds**. (b) Depiction of aligned set of molecules and 3D descriptors in cubic grid.

The dataset of 45 thiazolyl-pyrazoline derivatives [30]and the template (Figure 1a) was drawn using Chemsketch (ACD/Chemsketch Freeware Version 12.01) These compounds were prepared by Vlife Engine module of Vlife MDS as described previously [9,27-29].

### Computation of force field

The 38 thiazolyl-pyrazoline derivatives along with their pIC50 (negative logarithm of IC50) values (Table S1; additional file 1) were used for calculation of force field as described previously [9,27-29].

### Building the 3D-QSAR model of thiazolyl-pyrazoline derived compounds

Sphere exclusion method was applied for division of 38 compounds comprising the dataset into training and test set. Stepwise forward multiple regression method was utilized using the advanced variable selection and model building wizard for development of the 3D-QSAR model with default values. The stepwise forward variable selection algorithm has been described in previous studies [9,27,28,31].

### Validation of the developed 3D-QSAR model

The integrity of the developed 3D-QSAR model was confirmed with the help of different statistical parameters including squared correlation coefficient (r^2^), cross validated squared correlation coefficient (q^2^), predicted squared correlation coefficient (pred_r^2^), F-test and standard error. The model is said to be robust if it has the following statistical parameters r^2 ^> 0.6, q^2 ^> 0.6 and pred_r^2 ^> 0.5 [32-34]. The F-test is described as the variance observed by the developed QSAR model divided by the variance due to the error in the regression. Hence, statistical significance of the developed model can be explained with high F-test. The low standard error of Pred_r^2^se, q^2^_se and r^2^_se showed absolute fitness quality of the model.

### Model cross-validation

The developed model was first validated internally and then externally as described previously [9,27,28,31]. Briefly internal validation was carried out using the leave-one-out (q^2^, LOO) method while external validation involved prediction of inhibitory activity of each molecule comprising the test set by means of the QSAR model generated using compounds in the training set.

Y randomisation test was employed for examining the robustness of the developed models for training sets by calculation of Z-score as described previously [9,27,28,31].

### Prediction of ZINC library using developed 3D-QSAR model

A natural compound ZINC database containing 0.2 million compounds was prepared and used for prediction of inhibitory activity using the developed 3D-QSAR model. Compounds with high predicted inhibitory activity were selected for docking analysis.

### Docking of top scoring compounds with EGFR

Docking of the top two compounds with WT and T854A structures was performed using the Glide module of Schrodinger [35,36] as described previously [13,25,37].

## Results and discussion

### Structural and functional analysis of EGFR tyrosine kinase domain upon mutation

**Figure 2:**
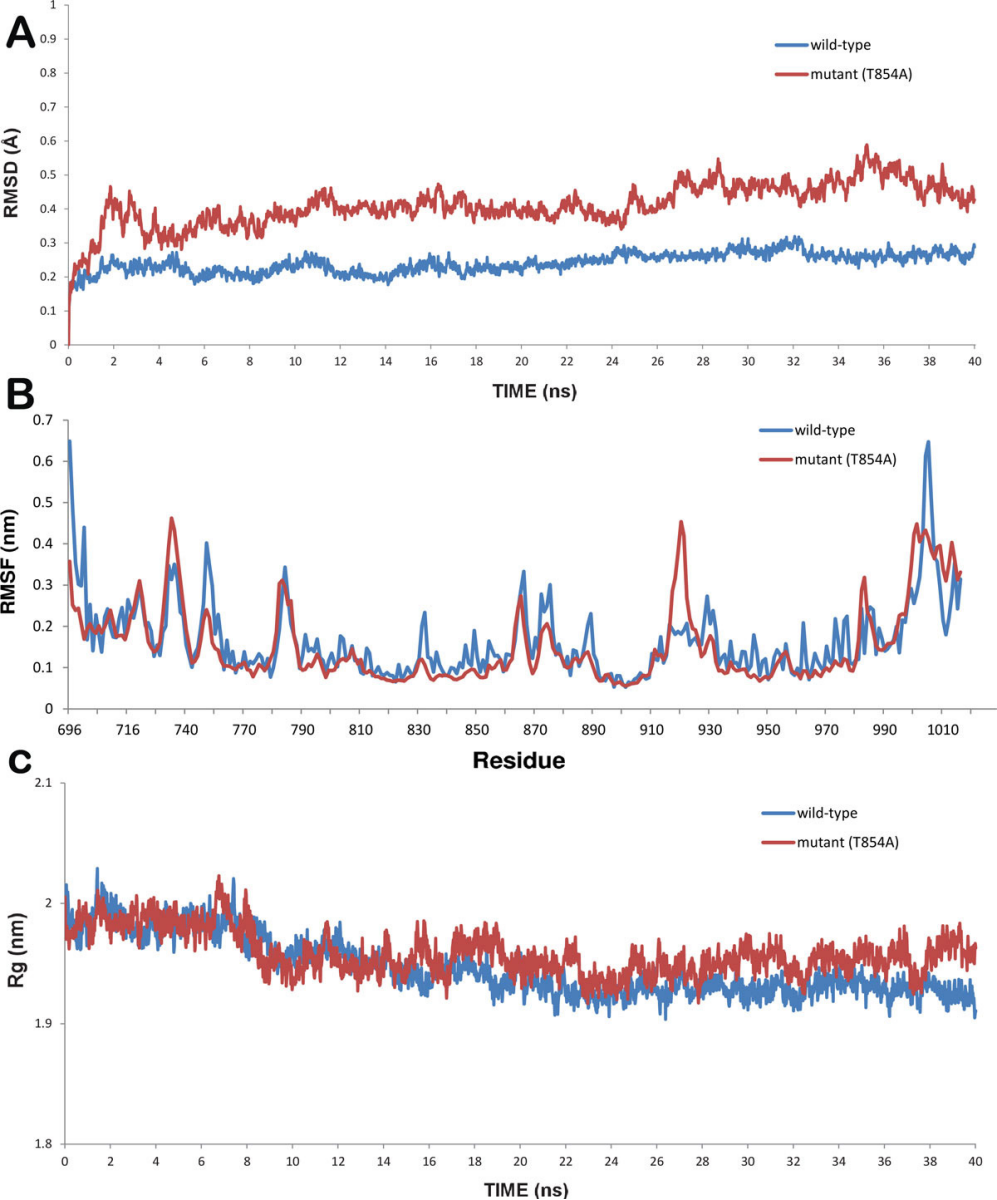
**Graphs showing (a) RMSD (b) RMSF and (c) Radius of gyration of wild-type (blue) and mutant (T854A) (red) protein**.

Molecular dynamics simulations for WT and mutant (T854A) EGFR TK protein was performed to gain insight into the structural and functional behaviour of the drug resistance associated mutation. We studied RMSD, RMSF, radius of gyration (Rg), solvent accessible surface area (SASA) and ED analysis between the WT and mutant (T854A) EGFR protein. RMSD for the backbone of the protein structures were calculated from the initial structure (Figure 2a). In this figure, till 14ns WT showed backbone RMSD of ∼0.13 to ∼0.27 nm during this part of simulations. After 14 ns WT structure exhibited minimum deviation till the end of simulation that is 40 ns with its backbone RMSD ranging from ∼0.14 to ∼0.22 nm, where as mutant structure showed maximum deviation till the end of simulation resulting backbone RMSD of ∼0.13 to ∼0.47 nm respectively. This stable RMSD provided a suitable basis for further analysis. For determining the effect of mutation on the behaviour of residues, the RMSF values of WT and mutant (T854A) structures were calculated (Figure 2b). The results indicate higher degree of flexibility in mutant (T854A) in comparison to WT.

Another parameter radius of gyration (Rg) defined as the mass-weight root mean square distance of collection of atoms from their common centre of mass was helpful in giving further insight into the structural changes due to mutation and the overall dimension of the protein. Plot of radius of gyration of protein vs. time is shown in Figure 2c. It can be seen that mutant (T854A) structure exhibited higher Rg value in comparison to WT structure. Variation of SASA for both WT and mutant (T854A) proteins with respect to time can be seen in Figure S1(a). WT structure was observed to have higher value of SASA with time, while mutant (T854A) showed lower value of SASA. Greater fluctuation in Rg in mutant (T854A) structure suggested structural alteration in the mutant structure. Since, hydrogen bonds play an important role in maintaining the stable conformation of protein, analysis of WT and mutant (T854A) proteins were performed with respect to time (Figure S1(b)). The total energy (Figure S1(c)) was observed to be more or less the same throughout the simulations for both WT and mutant (T854A).

All these results indicate that mutation (T854A) rendered the protein structure more flexible affecting the structural and functional behaviour of EGFR TK protein. This result was further validated by principal component analysis (PCA) analysis.

**Figure 3:**
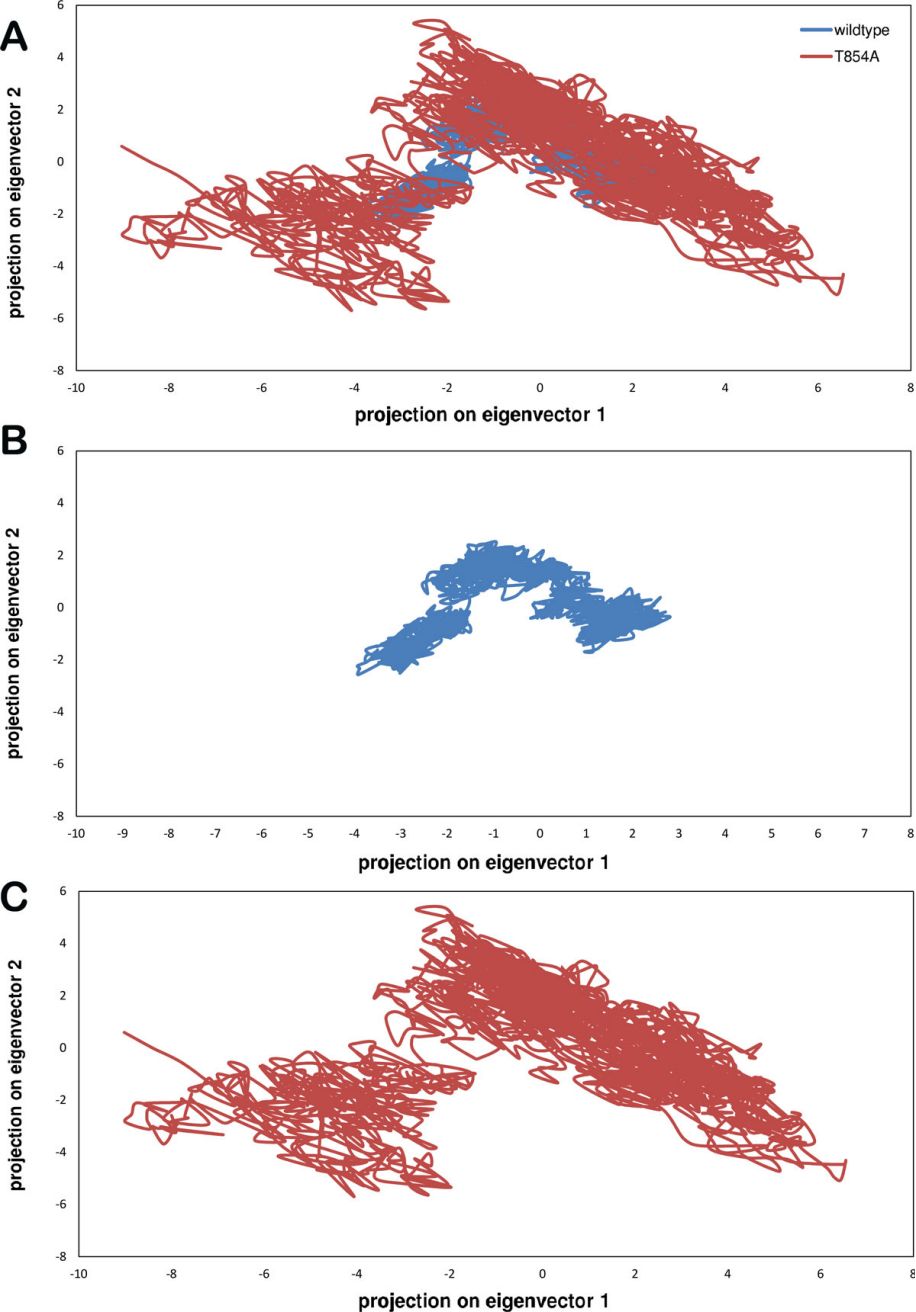
**(a) Projection of the motion of the protein in phase space along the first two principal eigenvectors of wild-type (blue) and mutant (T854A) (red) protein structures**.

Essential dynamics (ED) analysis gives an improved analysis of dynamical mechanical properties of the protein system. To further support our MD simulations results, the large-scale collective motions of the WT and mutant (T854A) protein using ED analysis were determined. Principal components are the eigenvectors of a covariance matrix. This projection gives the change of particular trajectory along each eigenvector. The range of the corresponding eigenvalues (Figure 3) indicated that the fluctuation of the protein system was basically restricted to the first two eigenvectors. The motion of the two proteins in phase space can be shown by the projection of trajectories obtained at 300 K onto the first two principal components (PC1, PC2) in which we observe clusters of stable states. Analysis of these plots reveals that the clusters are well defined in WT than mutant (T854A). Also, mutant (T854A) covers a greater region of phase space mainly along PC1 plane than WT. It can thus be said that mutant (T854A) is more flexible than WT at 300 K. The values for trace of the diagonalized covariance matrix of the Cα atomic positional fluctuations obtained for WT protein and mutant (T854A) protein were 7.603 nm^2 ^and 18.3734 nm^2 ^respectively. Trace is the total variance of the dataset thus again confirming the overall increased flexibility of mutant than WT at 300 K.

**Figure 4:**
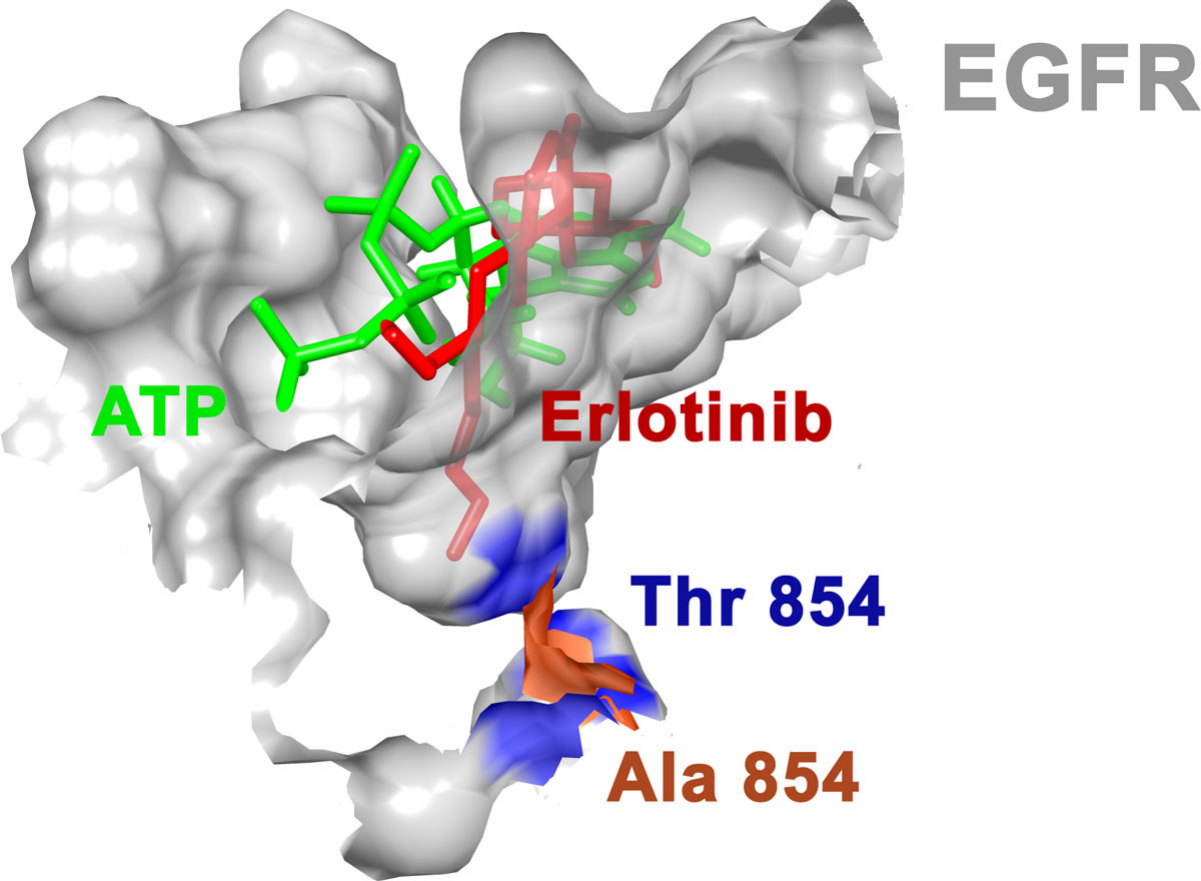
**Change in erlotinib binding site due to T854A mutation**.

Also it can be seen in Figure 4, T854A mutation causes change in active site. Since, 854 lies in the contact region of erlotinib but not close to ATP, this mutation results in reduced affinity for erlotinib while maintaining its kinase activity. Substitution of alanine in place of threonine causes the binding surface to move away from erlotinib indicating a possible reason for its decreased binding affinity. This mutation causes flexibility in the binding region of erlotinib while not affecting binding of ATP thus explaining its acquired resistance and maintained functionality.

### 3D QSAR model data selection

A 3D-QSAR model development gives a statistical relationship between the structures and activity of chemical compounds by calculation of 3D molecular descriptors involving steric, electrostatic and hydrophobic points marked on the 3D spatial grid. The invariable columns were removed after computing the force field grid descriptors which resulted in 3163 descriptors from 3268 descriptors, thus removing 105 invariable descriptors. For development of the QSAR model, pIC50 was chosen as the dependent variable while the calculated 3D descriptors as independent variable. Division of dataset resulted in 11 compounds in test set while the rest 27 compounds in training set. The test set consisted of compounds 6, 9, 12, 28, 29, 32, 36, 37, 40, 44 and 45.

### 3D-QSAR model development and validation

Stepwise forward (SW) multiple regression (MR) method was applied for development of 3D-QSAR model. The descriptors chosen were E_337, S_335, E_832, E_424, S_151 and E_721 belonging to steric and electrostatic field energy of interactions with the numbers representing their respective spatial grid points. In this model, no hydrophobic descriptors were selected in the final model. The 3D QSAR model obtained is:

$$\begin{array}{c} pIC50=\left[0.2989\left(\pm 0.0020\right)\times E\text{\_}337\right]+\left[3.2763\left(\pm 0.5560\right)\times S\text{\_}335\right]\\ +\left[0.1785\left(\pm 0.0003\right)\times E\text{\_}832\right]+\left[0.4938\left(\pm 0.0033\right)\times E\text{\_}424\right]\\ -\left[11.7460\left(\pm 0.3402\right)\times S\text{\_}151\right]-\left[0.6486\left(\pm 0.0019\right)\times E\text{\_}721\right]\\ +5.0198 \end{array}$$

Each descriptor is associated with a numerical coefficient and its error while the last single numerical value is the regression coefficient. Internal and external validation of the developed model was carried out using the LOO method by calculating statistical parameters and meeting critical requirements for a model to be robust. The statistical parameters obtained for this model included correlation coefficient r^2 ^(0.9751), cross-validated correlation coefficient q^2 ^(0.9491), predicted correlation coefficient pred_r^2 ^(0.9525), low standard error value, r^2^_se (0.0966), q^2^_se (0.1380) andpred_r^2^_se (0.1282) which confirm the model to be robust. Along with this, high value of F-test (130.3822) implied that the developed QSAR model is 99% statistically valid with 1 in 10000 chance of failure. There are other important statistical parameters such as Z-scores for r^2^, q^2 ^and pred_r^2 ^which are also important for QSAR model validation. Zscore_r^2 ^of 6.7926 implies a 100%area under the normal curve, Zscore_q^2 ^of 4.3671 implies a 99.99% area under the normal curve and Zscore_pred_r^2 ^of 1.6521 implies a 95.0743% area under the normal curve. These percentages indicate that the respective scores are near the mean 'μ' thus validating the model's statistical robustness. A parameter p-value for each of r^2^, q^2 ^and pred_r^2 ^was also obtained to be statistically significant with values 0.0001, 0.0001 and 0.09 respectively.

**Figure 5:**
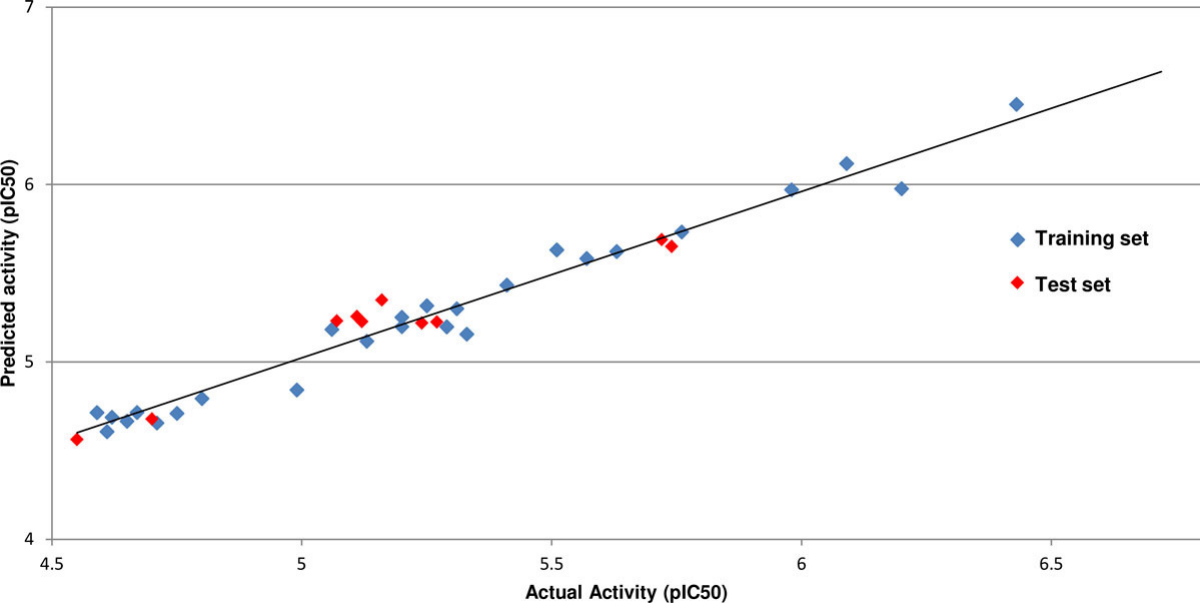
**Graph of observed versus predicted activity for training and test set**.

The robustness of the model can also be validated by radar and fitness plots. The fitness plot (Figure 5a) shows the extent of variation between the actual and predicted inhibitory activities of the thiazolyl-pyrazoline derived compounds. The radar plots (Figure S2 (a,b); Additional file 1) express the quality of the 3D-QSAR model by the extent of overlap between the actual value (blue) and predicted activity (red) lines. The contribution plot for each descriptor (Figure S2(c); additional file 1) specifies contribution of the properties that should be present in the lead compound for improving its inhibitory activity. Descriptors with positive contribution enhance the inhibitory activity of the lead compound whereas those with negative contribution reduce the same. Positive contribution for electrostatic descriptor shows a requirement of electropositive group at the substitution site and an electronegative group in case of negativeshi contribution.

The grid points E_337, E_832, S_335 and E_424 had a positive contribution (8.087%, 17.767%, 5.291% and 13.366% respectively) in the developed 3D-QSAR model against EGFR, while the descriptors S_151 and E_463 show negative contribution of 24.048% and 31.442% respectively. The grid points can be seen in Figure 1b. Steric descriptors represent the class of bulk descriptors which describe both size and shape of the molecules and fragments. Thus, positive contribution of a steric descriptor at specific grid point indicates the importance of a bulky group at that position. The value for each descriptor and predicted inhibitory activity for the dataset is mentioned in Table S2 (additional file 1).

The second class of descriptors, electrostatic descriptors give the importance of electronegative and electropositive groups at a particular site. Electrostatic descriptors with positive contribution imply the significance of presence of electropositive groups while those with negative contribution signify the importance of presence of electronegative groups.

### Activity prediction of ZINC libraries using developed 3D-QSAR model

**Figure 6:**
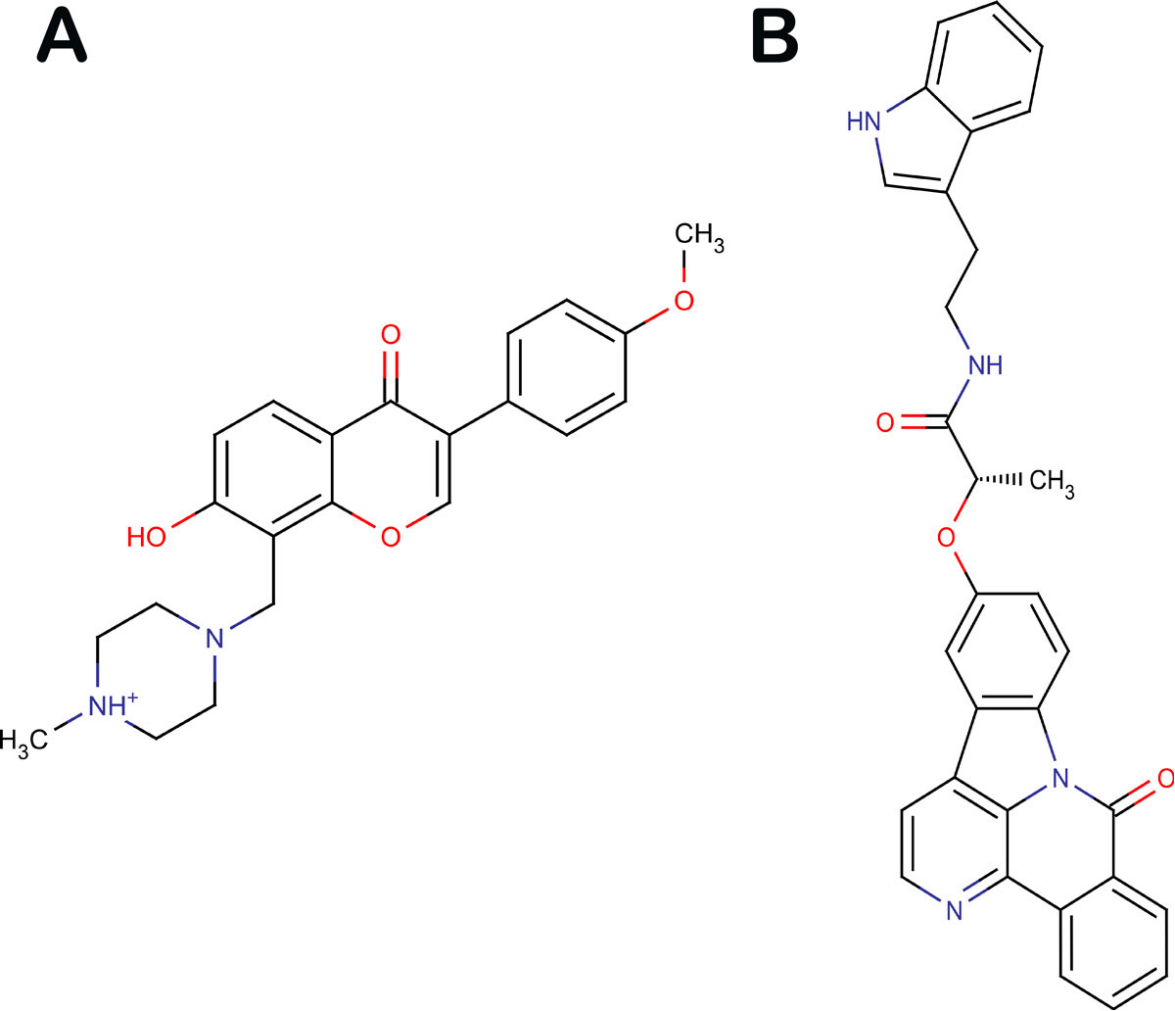
**Structure of top (a) HCO and (b) NOP**.

A total of 0.2 million natural compounds from ZINC library were screened and the highest predicted activity was observed to be 13.436 with 195 compounds having predicted activity above 8 and extrapolation between -1 and 1. We report the top two compounds with highest predicted activity. The first compound *7-hydroxy-3-(4-methoxyphenyl)-8-[(4-methylpiperazin-1-yl)methyl]-4H-chromen-4-one *[ZINC ID: 20391511] (HCO) had a predicted activity (pIC50) of 13.44 while the second compound *N-(2-(1H-indol-3-yl)ethyl)-2-((8-oxo-8H-benzo[c]indolo[3,2,1-ij][1, 5]naphthyridin-12-yl)oxy)propanamide *[ZINC ID: 08792354] (NOP) possessed a predicted activity value of 11.92 (Figure 6). The QSAR model generated was also used to predict the inhibitory activity of a second generation drug, BIBW2992, as reported by Bean et al as a positive control [8]. It was observed that HCO and NOP possessed better predicted inhibitory activity than BIBW2992 (4.3). Values of top 10 ZINC compounds with their predicted activity can be seen in Table 1.

**Table 1:** Predicted activity value (pIC50) of top ten ZINC compounds.

**S.No**.	ZINC ID	Predicted Activity	Extrapolation
1	ZINC20391511	13.436	-0.22
2	ZINC08792354	11.92	0.104
3	ZINC34105774	11.075	0.232
4	ZINC12892580	9.957	-0.373
5	ZINC11865797	9.883	0.314
6	ZINC68604752	9.68	-0.364
7	ZINC08877152	9.513	0.34
8	ZINC70700724	9.295	-0.051
9	ZINC33832195	9.142	-0.462
10	ZINC41669357	8.92	0.342

### Docking analysis of HCO and NOP to both WT and T854A structures

Both compounds (HCO and NOP) with highest predicted inhibitory activity against WT were docked with WT and T854A structures. The first compound HCO showed a binding affinity of -13.025 kJ/mol with WT while showing a better binding affinity of -16.485 kJ/mol with T854A structure. The second compound NOP also showed a better binding affinity to T854A (-8.598 kJ/mol) than WT (-8.037 kJ/mol). The results are summarised in table 2. Thus these compounds can be considered as lead compounds against both WT and T854A structures.

**Table 2:** Binding affinity of HCO and NOP with WT and T854A mutant structures.

Compound	Score (kJ/mol)	
	WT	T854A
HCO	-13.025	-16.485
NOP	-8.037	-8.598

## Conclusion

In the present study, we performed molecular dynamics simulations on both wild-type (WT) and mutant (T854A) structures of EGFR to analyse the structural changes brought about by missense SNP resulting in T854A mutation. A 3D-QSAR model was developed using 38 thiazolyl-pyrazoline derivatives against WT which was then used to screen ZINC libraries by predicting their inhibitory activity (pIC50). The top two compounds were docked against WT and T854A structures. These compounds can be considered as lead drug candidates against both WT and mutant (T854A). The results indicate stability loss observed in RMSD, RMSF, Rg and SASA analysis. Thus it can be said that WT structure becomes more flexible upon mutation (T854A) which brings about changes in the binding site of erlotinib thus reducing its binding affinity and rendering the mutated protein to become drug resistant while maintaining its functionality. This was further supported by results obtained in PCA analysis. This generates the need to develop drugs that inhibit both WT and mutant proteins. We report two novel compounds (HCO and NOP) which have high predicted inhibitory activity against WT and high binding affinity against both WT and T854A mutant structure. Since these compounds possess better predicted inhibitory activity than BIBW2992 a known second-generation EGFR inhibitor for T854A change, they can be considered for further experimental validation as potent lead compounds. We present a comprehensive view of the correlation between the structure and inhibitory activity of thiazolyl-pyrazoline derived molecules. This study advances the use of thiazolyl-pyrazoline moiety as anti-cancer. Results of this study will also prove to be useful in designing potent anti-tumorals based on EGFR TK inhibition to further develop drugs against cancer.

## Competing interests

The authors declare that they have no competing interests.

## Authors' contributions

SG, SJ and AS designed the methods and experimental setup. SG and SJ carried out the work. All the four authors wrote the manuscript.

## Supplementary Material

Additional file 1 This file includes the following figures and tables. **Figure S1: **Graphs showing (a) solvent accessible surface area (SASA) (b) Hydrogen bonds and (c) Total energy of wild-type (blue) and mutant (T854A) (red) protein. **Figure S2: **Depicting radar plots for (a) training set (b) test set and (c) contribution plot for 3D descriptors. **Table S1: **Details of thiazolyl-pyrazoline derived compounds along with their actual activity value against WT EGFR. **Table S2: **Values for descriptors and predicted activity value of thiazolyl-pyrazoline derivatives. Click here for file

## Declarations

AG would like to acknowledge financial support from Department of Science and Technology, Government of India towards publication of this article.

This article has been published as part of *BMC Genomics *Volume 16 Supplement 5, 2015: Proceedings of the 10th International Conference of the Brazilian Association for Bioinformatics and Computational Biology (X-Meeting 2014). The full contents of the supplement are available online at http://www.biomedcentral.com/bmcgenomics/supplements/16/S5.
