# Supplementary material for: Structural investigations of T854A mutation in EGFR and identification of novel inhibitors using structure activity relationships
Source: BMC Genomics. 2015 May 26;16(Suppl 5):S8. doi: 10.1186/1471-2164-16-S5-S8 (PMC4460657; doi:10.1186/1471-2164-16-S5-S8)
Supplement: Additional file 1 — This file includes the following figures and tables. Figure S1: Graphs showing (a) solvent accessible surface area (SASA) (b) Hydrogen bonds and (c) Total energy of wild-type (blue) and mutant (T854A) (red) protein. Figure S2: Depicting radar plots for (a) training set (b) test set and (c) contribution plot for 3D descriptors. Table S1: Details of thiazolyl-pyrazoline derived compounds along with their actual activity value against WT EGFR. Table S2: Values for descriptors and predicted activity value of thiazolyl-pyrazoline derivatives. [file 1471-2164-16-S5-S8-S1.docx]

**Additional file - 1**

**Figure S1:** Graphs showing (a) solvent accessible surface area (SASA) (b) Hydrogen bonds and (c) Total energy of wild-type (blue) and mutant (T854A) (red) protein.

**
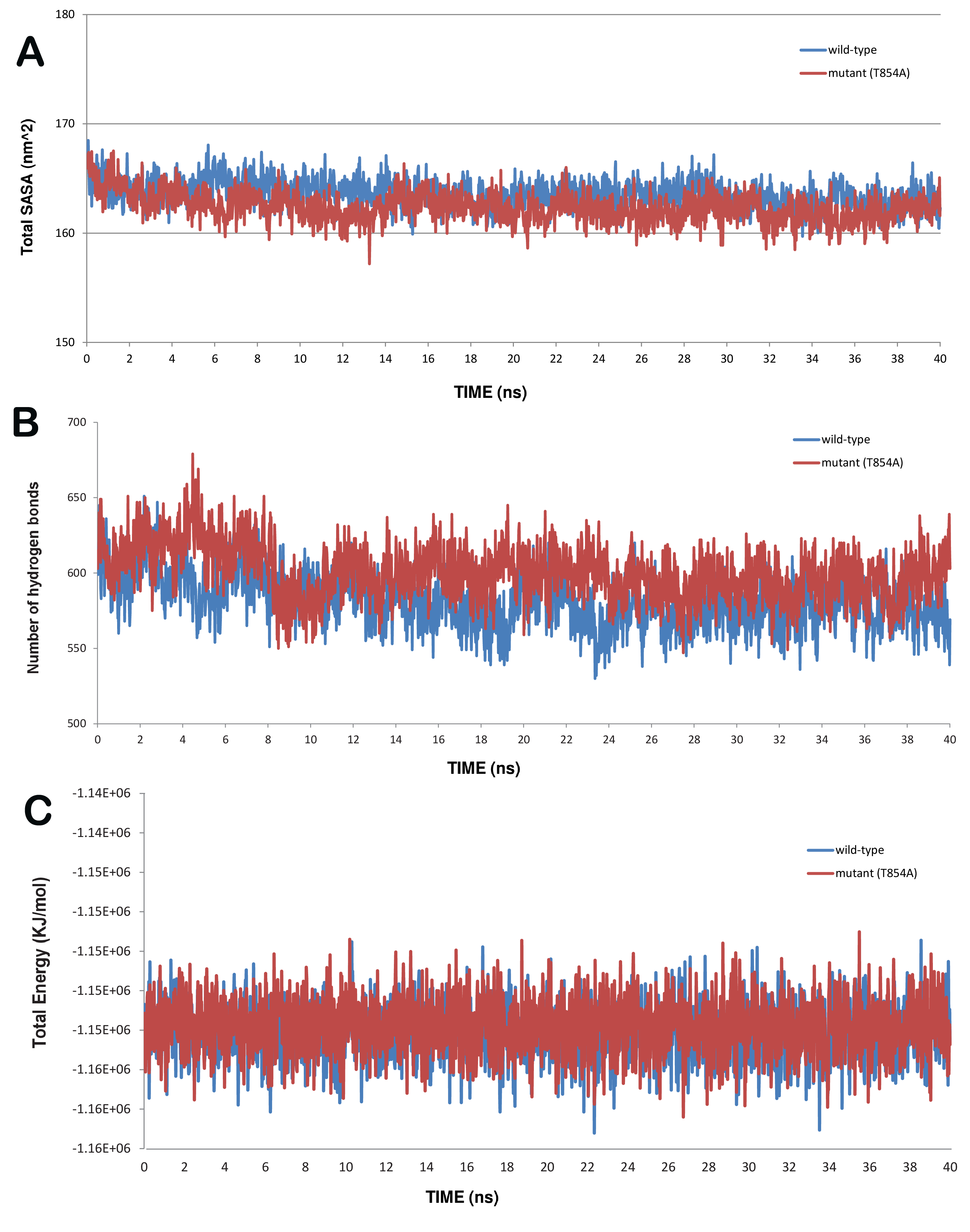
**

**Figure S2:** Depicting radar plots for (a) training set (b) test set and (c) contribution plot for 3D descriptors.


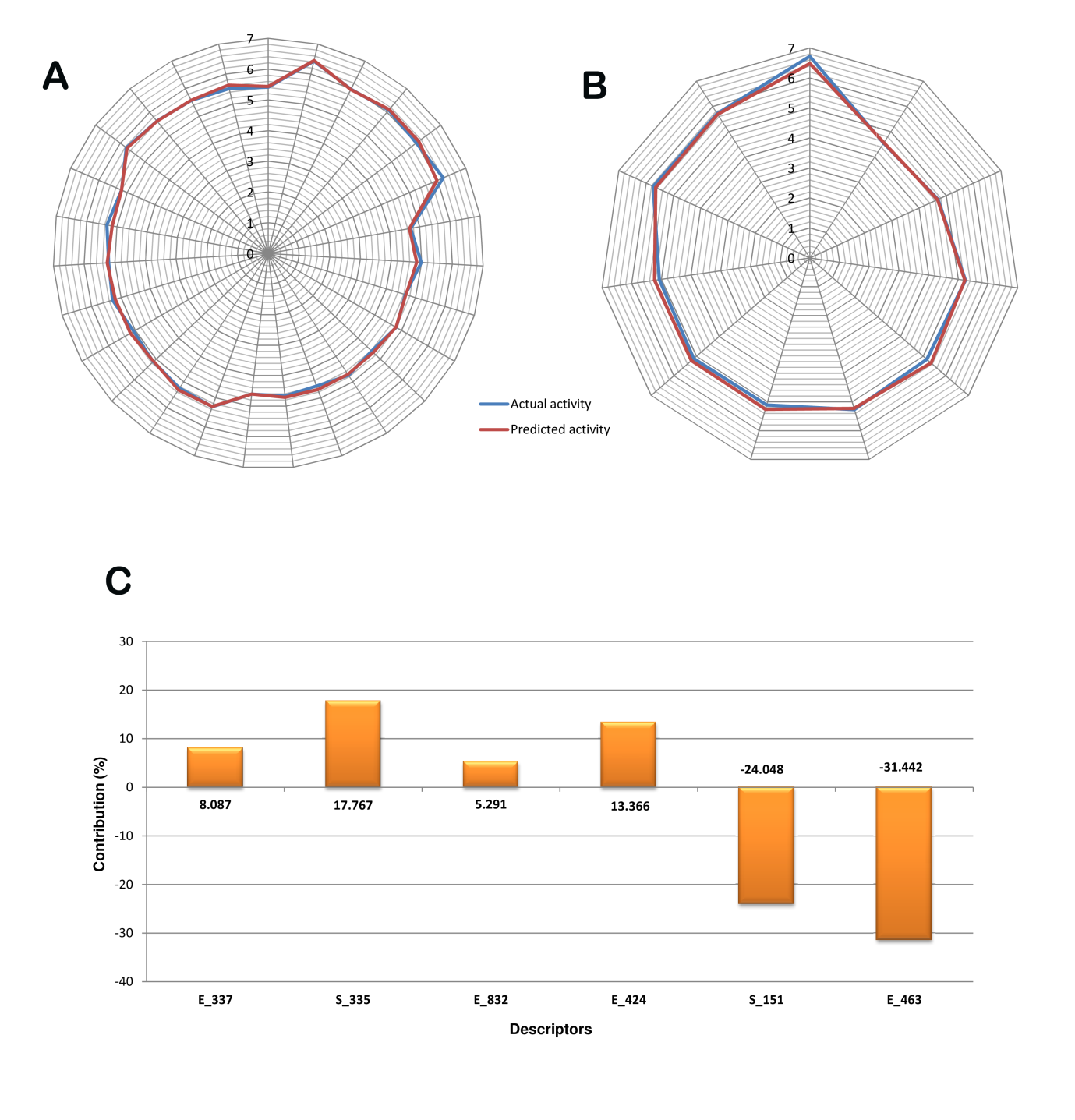


**Table S1:** Details of thiazolyl-pyrazoline derived compounds along with their actual activity value against WT EGFR.

| **S.No.** | **Compound No.** | **Structure** | **IC50 (µM)** | **pIC50** |
| --- | --- | --- | --- | --- |
| 1 | 10 | 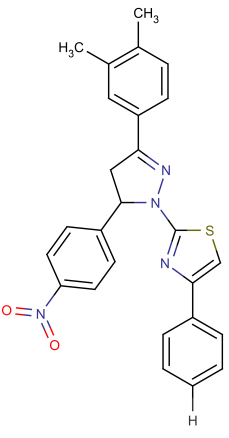 | 3.85 | 5.414539 |
| 2 | 12 | 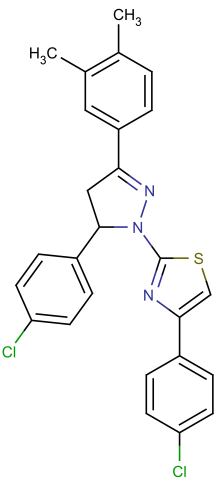 | 0.19 | 6.721246 |
| 3 | 13 | 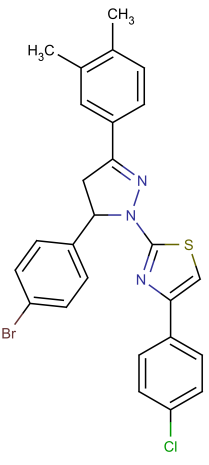 | 0.37 | 6.431798 |
| 4 | 14 | 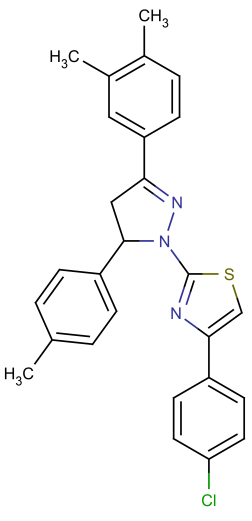 | 1.04 | 5.982967 |
| 5 | 15 | 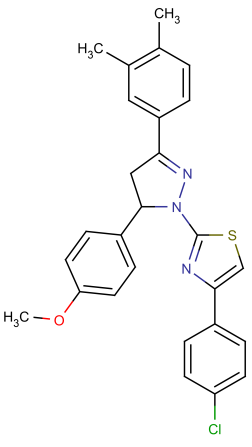 | 0.82 | 6.086186 |
| 6 | 16 | 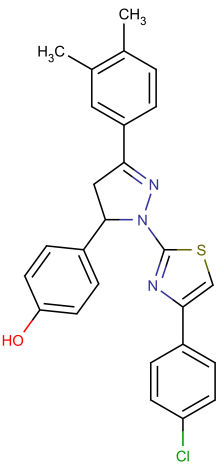 | 0.97 | 6.013228 |
| 7 | 17 | 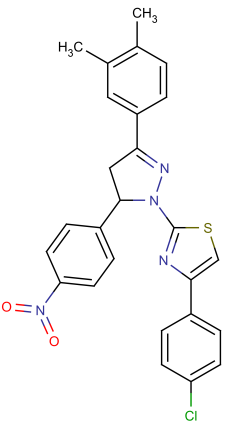 | 0.63 | 6.200659 |
| 8 | 21 | 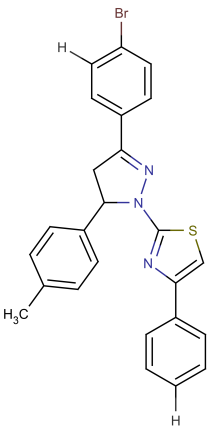 | 19.64 | 4.706859 |
| 9 | 22 | 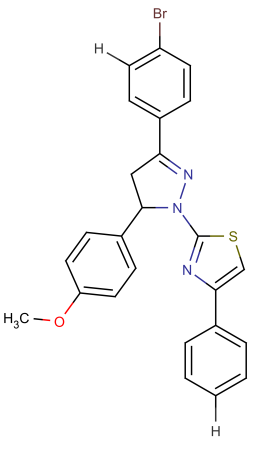 | 10.17 | 4.992679 |
| 10 | 23 | 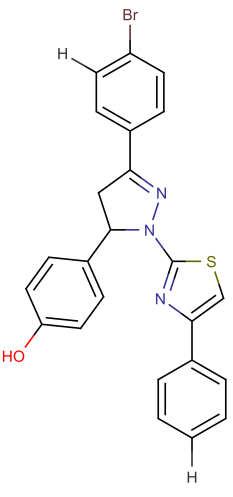 | 22.36 | 4.650528 |
| 11 | 24 | 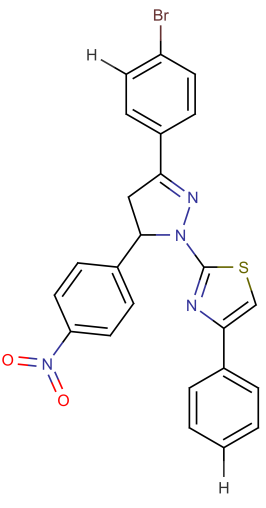 | 15.72 | 4.803547 |
| 12 | 25 | 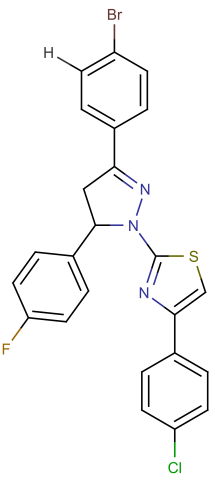 | 24.16 | 4.616903 |
| 13 | 26 | 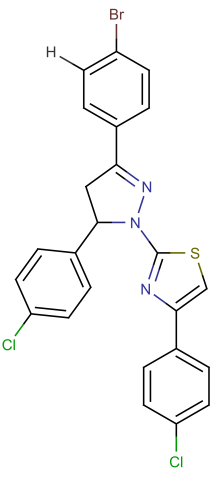 | 17.84 | 4.748605 |
| 14 | 27 | 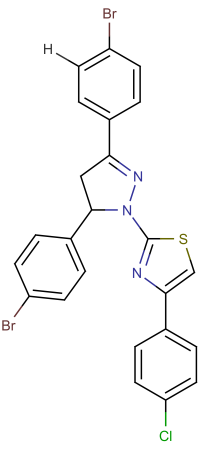 | 25.74 | 4.589391 |
| 15 | 28 | 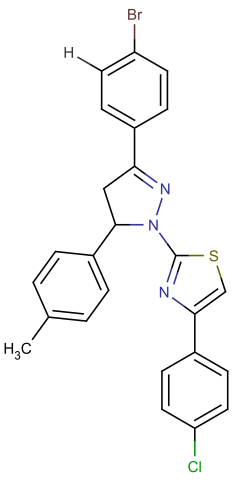 | 28.17 | 4.550213 |
| 16 | 29 | 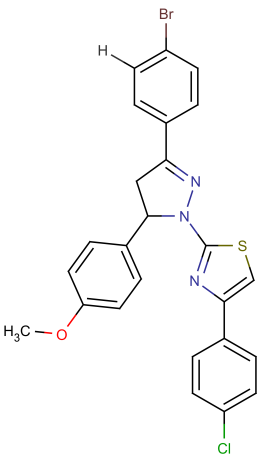 | 19.83 | 4.702677 |
| 17 | 30 | 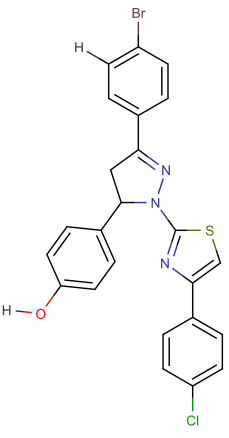 | 21.52 | 4.667158 |
| 18 | 31 | 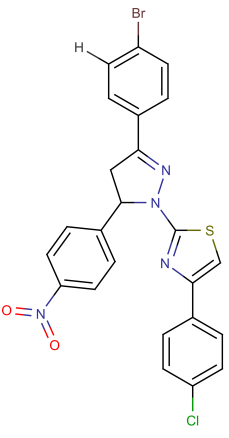 | 24.37 | 4.613144 |
| 19 | 32 | 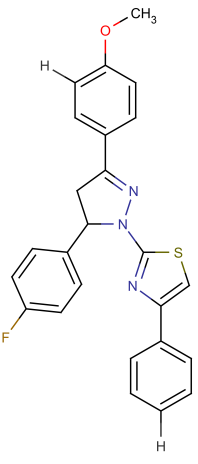 | 5.71 | 5.243364 |
| 20 | 33 | 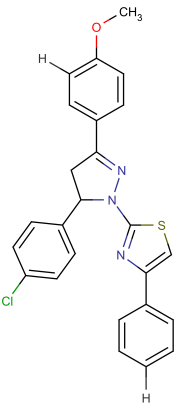 | 4.94 | 5.306273 |
| 21 | 34 | 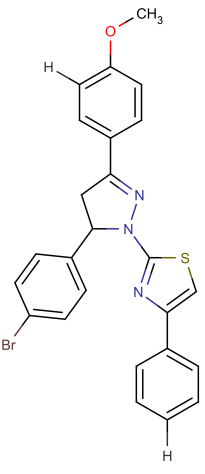 | 5.56 | 5.254925 |
| 22 | 35 | 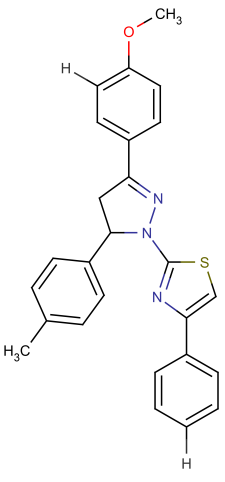 | 7.48 | 5.126098 |
| 23 | 36 | 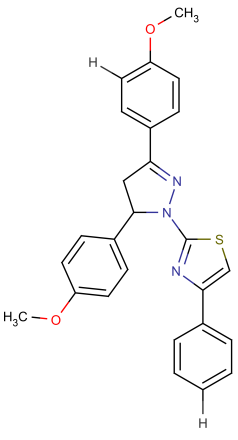 | 6.92 | 5.159894 |
| 24 | 37 | 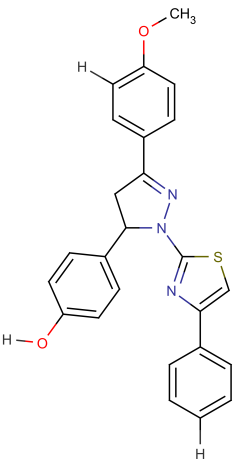 | 5.35 | 5.271646 |
| 25 | 38 | 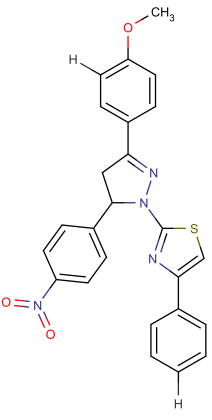 | 8.63 | 5.063989 |
| 26 | 39 | 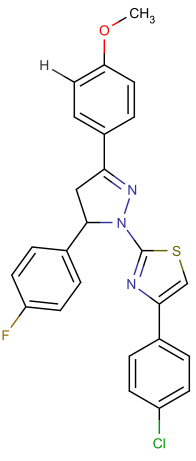 | 5.07 | 5.294992 |
| 27 | 40 | 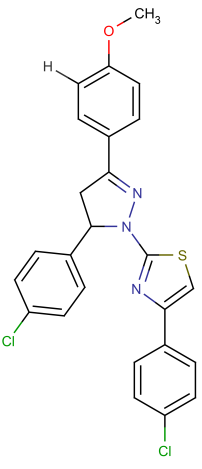 | 7.78 | 5.10902 |
| 28 | 41 | 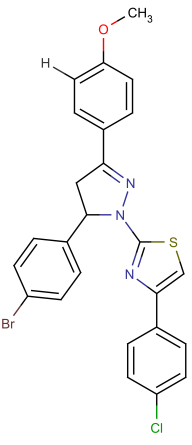 | 6.31 | 5.199971 |
| 29 | 42 | 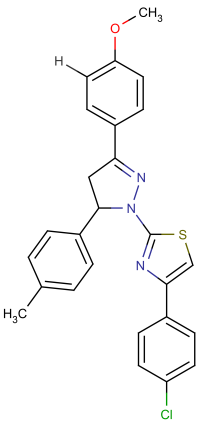 | 4.68 | 5.329754 |
| 30 | 43 | 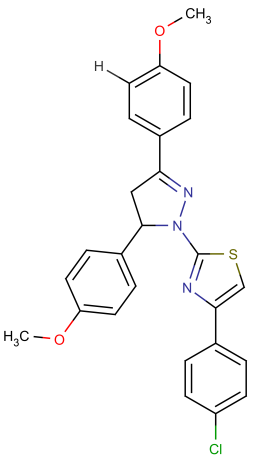 | 6.27 | 5.202732 |
| 31 | 44 | 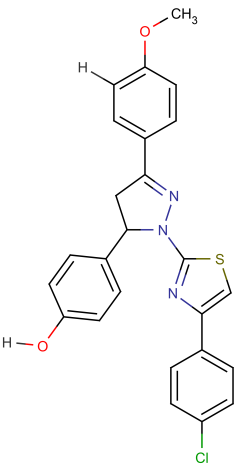 | 7.66 | 5.115771 |
| 32 | 45 | 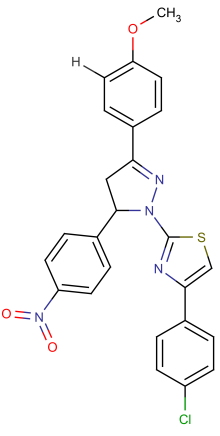 | 8.43 | 5.074172 |
| 33 | 4 | 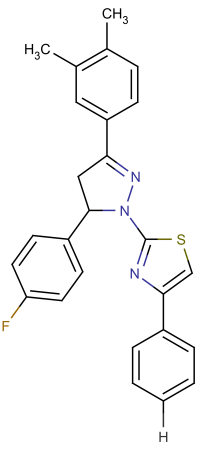 | 1.73 | 5.761954 |
| 34 | 5 | 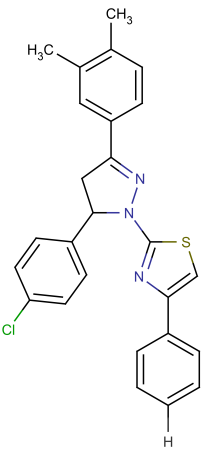 | 2.36 | 5.627088 |
| 35 | 6 | 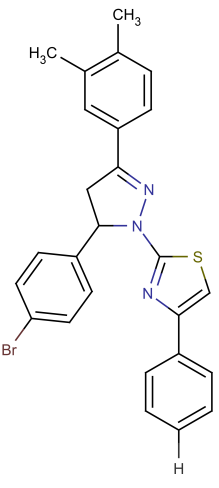 | 1.84 | 5.735182 |
| 36 | 7 | 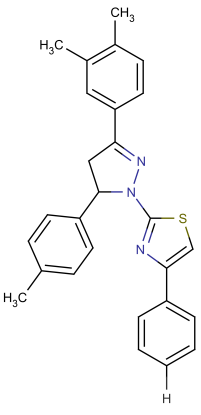 | 2.67 | 5.573489 |
| 37 | 8 | 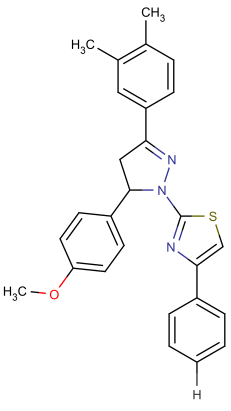 | 3.08 | 5.511449 |
| 38 | 9 | 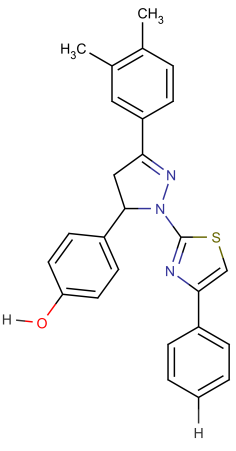 | 1.92 | 5.716699 |

**Table S2:** Values for descriptors and predicted activity value of thiazolyl-pyrazoline derivatives.

| **S.No.** | **Compound No.** | **Actual pIC50** | **S_151** | **E_337** | **E_721** | **S_335** | **E_832** | **E_424** | **Predicted pIC50** |
| --- | --- | --- | --- | --- | --- | --- | --- | --- | --- |
| 1 | 10 | 5.41 | -0.293 | 0.638 | 0.7 | -0.88 | 0.229 | 0.419 | 5.568 |
| 2 | 12 | 6.72 | -0.305 | 0.798 | 0.471 | -0.826 | -0.261 | 0.349 | 5.953 |
| 3 | 13 | 6.43 | -0.307 | 0.796 | 0.428 | -0.845 | -0.342 | 0.323 | 5.921 |
| 4 | 14 | 5.98 | -0.305 | 0.722 | 0.277 | -0.899 | -0.687 | 0.238 | 5.686 |
| 5 | 15 | 6.09 | -0.291 | 0.703 | 0.314 | -0.746 | -0.908 | 0.339 | 6.012 |
| 6 | 16 | 6.01 | -0.305 | 0.844 | 0.546 | -0.828 | -0.277 | 0.351 | 5.908 |
| 7 | 17 | 6.2 | -0.306 | 0.865 | 0.804 | -0.894 | 0.318 | 0.477 | 5.713 |
| 8 | 21 | 4.71 | -0.109 | 0.65 | 0.233 | -0.712 | -0.769 | 0.331 | 4.033 |
| 9 | 22 | 4.99 | -0.101 | 0.818 | 0.357 | -0.722 | -0.805 | 0.466 | 3.947 |
| 10 | 23 | 4.65 | -0.105 | 0.782 | 0.471 | -0.732 | -0.446 | 0.471 | 3.933 |
| 11 | 24 | 4.8 | -0.093 | 0.893 | 0.806 | -0.68 | 0.287 | 0.599 | 3.973 |
| 12 | 25 | 4.62 | -0.106 | 0.814 | 0.7 | -0.713 | 0.028 | 0.57 | 4.008 |
| 13 | 26 | 4.75 | -0.1 | 0.801 | 0.573 | -0.723 | -0.192 | 0.517 | 3.909 |
| 14 | 27 | 4.59 | -0.099 | 0.788 | 0.525 | -0.723 | -0.281 | 0.496 | 3.906 |
| 15 | 28 | 4.55 | -0.103 | 0.713 | 0.339 | -0.722 | -0.666 | 0.397 | 3.929 |
| 16 | 29 | 4.7 | -0.114 | 0.695 | 0.382 | -0.651 | -0.869 | 0.485 | 4.277 |
| 17 | 30 | 4.67 | -0.107 | 0.765 | 0.515 | -0.715 | -0.343 | 0.496 | 4.011 |
| 18 | 31 | 4.61 | -0.096 | 0.915 | 0.889 | -0.712 | 0.381 | 0.637 | 3.896 |
| 19 | 32 | 5.24 | -0.209 | 0.675 | 0.616 | -0.749 | -0.037 | 0.454 | 5.041 |
| 20 | 33 | 5.31 | -0.206 | 0.668 | 0.481 | -0.768 | -0.268 | 0.4 | 4.966 |
| 21 | 34 | 5.25 | -0.208 | 0.645 | 0.426 | -0.764 | -0.356 | 0.37 | 4.999 |
| 22 | 35 | 5.13 | -0.206 | 0.589 | 0.258 | -0.768 | -0.724 | 0.283 | 4.944 |
| 23 | 36 | 5.16 | -0.208 | 0.72 | 0.359 | -0.772 | -0.78 | 0.402 | 4.973 |
| 24 | 37 | 5.27 | -0.209 | 0.627 | 0.43 | -0.753 | -0.417 | 0.381 | 5.029 |
| 25 | 38 | 5.06 | -0.199 | 0.812 | 0.803 | -0.783 | 0.307 | 0.531 | 4.831 |
| 26 | 39 | 5.29 | -0.207 | 0.729 | 0.705 | -0.753 | 0.054 | 0.508 | 5.007 |
| 27 | 40 | 5.11 | -0.2 | 0.733 | 0.58 | -0.774 | -0.172 | 0.466 | 4.879 |
| 28 | 41 | 5.2 | -0.199 | 0.737 | 0.541 | -0.781 | -0.246 | 0.447 | 4.84 |
| 29 | 42 | 5.33 | -0.206 | 0.589 | 0.327 | -0.74 | -0.654 | 0.337 | 5.031 |
| 30 | 43 | 5.2 | -0.211 | 0.609 | 0.384 | -0.69 | -0.828 | 0.426 | 5.234 |
| 31 | 44 | 5.12 | -0.205 | 0.682 | 0.522 | -0.75 | -0.323 | 0.441 | 4.996 |
| 32 | 45 | 5.07 | -0.194 | 0.892 | 0.908 | -0.767 | 0.399 | 0.6 | 4.824 |
| 33 | 4 | 5.76 | -0.288 | 0.626 | 0.54 | -0.813 | -0.121 | 0.372 | 5.742 |
| 34 | 5 | 5.63 | -0.292 | 0.558 | 0.402 | -0.853 | -0.344 | 0.308 | 5.65 |
| 35 | 6 | 5.74 | -0.292 | 0.541 | 0.35 | -0.849 | -0.434 | 0.286 | 5.665 |
| 36 | 7 | 5.57 | -0.291 | 0.499 | 0.176 | -0.838 | -0.803 | 0.198 | 5.68 |
| 37 | 8 | 5.51 | -0.293 | 0.61 | 0.273 | -0.869 | -0.873 | 0.308 | 5.618 |
| 38 | 9 | 5.72 | -0.29 | 0.596 | 0.362 | -0.843 | -0.49 | 0.297 | 5.667 |
